# Optimization of the Electrochemical Performance of a Composite Polymer Electrolyte Based on PVA-K_2_CO_3_-SiO_2_ Composite

**DOI:** 10.3390/polym13010092

**Published:** 2020-12-28

**Authors:** Bashir Abubakar Abdulkadir, John Ojur Dennis, Yas Al-Hadeethi, Muhammad Fadhlullah Bin Abd. Shukur, E. M. Mkawi, Nuha Al-Harbi, K. H. Ibnaouf, O. Aldaghri, Fahad Usman, Abdullahi Abbas Adam

**Affiliations:** 1 Fundamental and Applied Sciences Department, Universiti Teknologi PETRONAS, Tronoh 32610, Malaysia; johndennis@utp.edu.my (J.O.D.); mfadhlullah.ashukur@utp.edu.my (M.F.B.A.S.); fahatu11@gmail.com (F.U.); ibneeabdullah234@gmail.com (A.A.A.); 2 Department of Physics, Faculty of Science, King Abdulaziz University, Jeddah 21589, Saudi Arabia; yalhadeethi@kau.edu.sa (Y.A.-H.); emrzog@kau.edu.sa (E.M.M.); nallhibi0001@stu.kau.edu.sa (N.A.-H.); 3 Physics Department, College of Science, Imam Mohammad Ibn Saud Islamic University (IMSIU), P.O. Box 5701, Riyadh 11432, Saudi Arabia; khiahmed@imamu.edu.sa (K.H.I.); odaghri@gmail.com (O.A.)

**Keywords:** composite polymer electrolyte, K_2_CO_3_, PVA, SiO_2_, plasticizer

## Abstract

Composite polymer electrolyte (CPE) based on polyvinyl alcohol (PVA) polymer, potassium carbonate (K_2_CO_3_) salt, and silica (SiO_2_) filler was investigated and optimized in this study for improved ionic conductivity and potential window for use in electrochemical devices. Various quantities of SiO_2_ in wt.% were incorporated into PVA-K_2_CO_3_ complex to prepare the CPEs. To study the effect of SiO_2_ on PVA-K_2_CO_3_ composites, the developed electrolytes were characterized for their chemical structure (FTIR), morphology (FESEM), thermal stabilities (TGA), glass transition temperature (differential scanning calorimetry (DSC)), ionic conductivity using electrochemical impedance spectroscopy (EIS), and potential window using linear sweep voltammetry (LSV). Physicochemical characterization results based on thermal and structural analysis indicated that the addition of SiO_2_ enhanced the amorphous region of the PVA-K_2_CO_3_ composites which enhanced the dissociation of the K_2_CO_3_ salt into K^+^ and CO_3_^2^^−^ and thus resulting in an increase of the ionic conduction of the electrolyte. An optimum ionic conductivity of 3.25 × 10^−^^4^ and 7.86 × 10^−^^3^ mScm^−^^1^ at ambient temperature and at 373.15 K, respectively, at a potential window of 3.35 V was observed at a composition of 15 wt.% SiO_2_. From FESEM micrographs, the white granules and aggregate seen on the surface of the samples confirm that SiO_2_ particles have been successfully dispersed into the PVA-K_2_CO_3_ matrix. The observed ionic conductivity increased linearly with increase in temperature confirming the electrolyte as temperature-dependent. Based on the observed performance, it can be concluded that the CPEs based on PVA-K_2_CO_3_-SiO_2_ composites could serve as promising candidate for portable and flexible next generation energy storage devices.

## 1. Introduction

The current trend to produce green energy and suitable energy storage is driven by the need to replace fossil fuels which have an adverse effect on the environment [1]. With increasing development in sustainable power sources from sun and wind as well as in electric and hybrid vehicles with low CO_2_ emanations, energy storage devices such as supercapacitors have become a major field of interest to researchers [2]. They have recently received increased attention due to their numerous advantages arising from occupying an important position in relation to specific power and specific energy compared to other energy storage devices such as capacitors, batteries, and fuel cells, and hence have the essential characteristics to satisfy the energy storage requirements of the present and the future [3]. Supercapacitors may be categorized into three types: electrode double layer capacitors (EDLCs), pseudocapacitors, and hybrid capacitors. All three types of supercapacitors consist of three main components namely electrode, separator, and electrolyte on which the performance of the electrochemical supercapacitors depends.

The electrolyte is a vital component providing the ionic conductivity and operating potential window of the supercapacitor [2]. Electrolytes used in supercapacitors commonly have two important functions: providing ions during charge and discharge process and preventing the electrodes from short-circuiting. Most of the earliest studies on supercapacitors mainly reported the utilization of liquid electrolytes in both laboratories and industries [4,5]. Liquid electrolytes are mostly composed of aqueous solutions of acids such as H_2_SO_4_, LiSO_4_, and KOH, which allows supercapacitors to reach higher conductivity values [6]. However, the utilization of liquid electrolytes has significant limitations such as environmental unfriendliness since it may be easily leach out, low life cycle, long charging time, and low voltage window [7]. Recently, with the advances in electrolyte research coupled with serious disadvantages in using liquid electrolytes, polymer electrolytes (PE) in the form of a solid or gel have been proposed and researched [5,8].

Polymer electrolytes (PE) are a promising substitute for the traditional liquid electrolytes due to their long cycle life, adaptability, high ionic conductivity, high chemical and mechanical dependability, wide potential window, sustainable, environmental friendliness, and leakage elimination possibility [9]. A polymer, salt, and filler composite could be an appropriate way to produce unique composite polymer electrolyte (CPEs) that are capable of higher electrochemical performance in energy storage devices. It has been established that when salt with low lattice energy such as K_2_CO_3_ is dissolved in a polymer matrix, CPEs are formed [10]. It is reported that polymers such as polyvinyl alcohol (PVA) are attractive for the synthesis of CPEs. PVA is a synthetic polymer produced through the hydrolysis of polyvinyl acetate. Vinyl ester obtained from polymers is also used with chloroacetate rather than acetate. Properties such as biocompatibility, semi-crystalline nature, environmental friendliness, non-toxicity, high solubility in water, excellent film-forming capacity, high dielectric constant, and high thermal stability (200 °C) make PVA prospective material for the polymer electrolytes [11]. The existence of polar group (–OH) on the PVA support is adequate to dissolve conducting salts. PVA is a hydrophilic polymer and features a high density of reactive chemical functional groups. These functional groups are substantial for mixing with conducting salt and a filler. The utilization of PVA as substantive material as a host polymer for electrolyte was reported earlier [12].

Solid polymer electrolyte (SPE) and gel polymer electrolyte (GPE) or CPEs have shown enhanced reliability and dependability over liquid electrolytes [5]. Among the polymer electrolytes, GPEs have the advantage of wide potential window and high ionic conductivity [13]. GPEs were reported to have ionic conductivity that is very similar to that of liquid electrolytes but with diminished spillage. Normally, the polymer framework in GPE holds the liquid electrolyte consolidating the generally high conductivity of liquid electrolytes with the strong characteristics of solid electrolytes. GPEs or CPEs may be used as a thin film as both an electrolyte and a separator with low internal corrosion, simple principles and modes of construction, and flexibility in packaging. However, the shortcomings of GPEs are frail mechanical quality, which will bring about internal short circuits, and a restricted scope of working temperature [14]. To solve these shortcomings and improve the weak mechanical strength and the low ionic conductivity of GPEs or CPEs, fillers are usually added [15]. The addition of fillers is reported to be one amongst the foremost approaches to enhance the mobility of ions or/and the interfacial interaction among ions and polar groups within the electrolytes [16]. The semi-crystalline nature of the polymer in CPE also presents difficulty for ion transport [17]. One amongst the foremost favorable ways of increasing the amorphous region of the polymer and enhancing the conductivity in CPE is blending the polymer with an appropriate filler such as SiO_2_. Moreover, the incorporation of filler can modify the polymeric nature of the electrolyte and enhanced conductivity. The selection of filler and its concentration was reported to influence film absorptivity and mechanical properties [18].

SiO_2_ as a filler is an attractive material for preparing CPEs because it can improve electrochemical performance of the material by providing large surface area and high conductivity and promoting good electrolyte–electrode interaction. More enhancements of these properties depend on how well the silica dispersed into the CPEs. Good dispersion of SiO_2_ particles often leads to morphology suitable for the enhancement of electrochemical performance. Secondly, addition of hydrophilic fumed silica with silanol (Si–OH) groups on its surface makes the diameter of an electrolyte much better [17]. Furthermore, SiO_2_ as a filler was reported to serve as a supporter to stand tunneling structures and to hold the liquid electrolyte. It also improves ionic conductivity of the composites by reducing the crystallinity of the polymer and introducing Lewis acid–base interactions between the polar surface groups of the inorganic filler and the electrolyte ionic species. In addition, SiO_2_ increases mechanical properties of the electrolytes and expands the cation transference number and interfacial stability between the electrolyte–electrode interfaces [18].

The addition of silica into the electrolyte was found to reduce glass transition temperature (*T_g_*) and melting temperature (*T_m_*) of the CPEs, which could be due to the interaction between the added silica and cation that leads to the increase in the ionic conductivities [17]. Shekibi and co-workers [19] reported that addition of silica powders into PEs has two benefits. The first benefit is to improve the conductivity, which is verified by experiments. The mechanism is that addition of silica particles will prevent the process of polymer crystallization at low temperatures so that the amorphous region in charge of ionic conductivity is preserved. Another benefit is that the stability of the interface between the electrolytes and the electrodes can be improved with the addition of silica particles. Polymer electrolytes with ceramic fillers, salts, ionic liquids, and other plasticizers are widely reported recently [17,18,19,20,21,22]. However, the study of silica (SiO_2_) as a filler in a polymer-salt composite of PVA-K_2_CO_3_ is yet to be reported. Therefore, the effects of SiO_2_ particles on the electrochemical properties of CPEs based on PVA-K_2_CO_3_ composites were investigated and are reported herein.

The objective of the present work is to report the preparation and characterization of CPEs based on PVA-K_2_CO_3_ composite with varying amounts of SiO_2_ as filler. The study includes optimization of the amount of SiO_2_ in PVA-K_2_CO_3_ composite for improved ionic conductivity and potential window. The various samples of CPEs of PVA-K_2_CO_3_ composite with varying amounts of SiO_2_ are evaluated using FTIR, FESEM, TGA, differential scanning calorimetry (DSC), electrochemical impedance spectroscopy (EIS), and linear sweep voltammetry (LSV) for their functional groups, morphology, thermal stability, *T_g_*, ionic conductivity, and potential window, respectively.

## 2. Materials and Methods

### 2.1. Material

PVA (hydrolyzed 99%), silica, and potassium carbonate (anhydrous) were obtained from Sigma-Aldrich (002008792-H) supplied by Avantis chemicals supply (Ipoh-Perak, Malaysia). All the chemicals were of analytical grade and used as received. For all the experiments’ preparation, deionized water was used.

### 2.2. Preparation of CPE Based on PVA-K_2_CO_3_-SiO_2_ Composite

PVA as host polymer, potassium carbonate (K_2_CO_3_) as salt, and silica (SiO_2_) as filler were used to prepare the CPEs. The electrolytes were prepared with the dissolution technique with varying amounts (ratios) of the filler. For the preparation of the non-filler CPEs, a fixed ratio of PVA and K_2_CO_3_ (70:30) was added into 20 mL of deionized water in a glass beaker. The two solutions were mixed and heated at 80 °C with continuous vigorous stirring using thermostat until the two solutions were completely dissolved to form a composite of PVA-K_2_CO_3_ as reported in our previous study [23]. For the preparation of CPEs containing filler, an appropriate amount of SiO_2_ was added to the prepared PVA-K_2_CO_3_ composites, heated at 80 °C continuously with vigorous stirring until homogeneous, clear, and viscous solution were obtained. The optimization study was conducted by varying the amount SiO_2_ while keeping the polymer-salt ratio constant as shown in Table 1. The resulting polymer electrolytes were coded as PKS0, PKS5, PKS10, PKS15, PKS20, and PKS25 for electrolyte incorporated with 0, 5, 10, 15, 20, and 25 wt.% of SiO_2_, respectively. All the samples were stored in a desiccator prior to analysis and testing.

**Figure 1:**
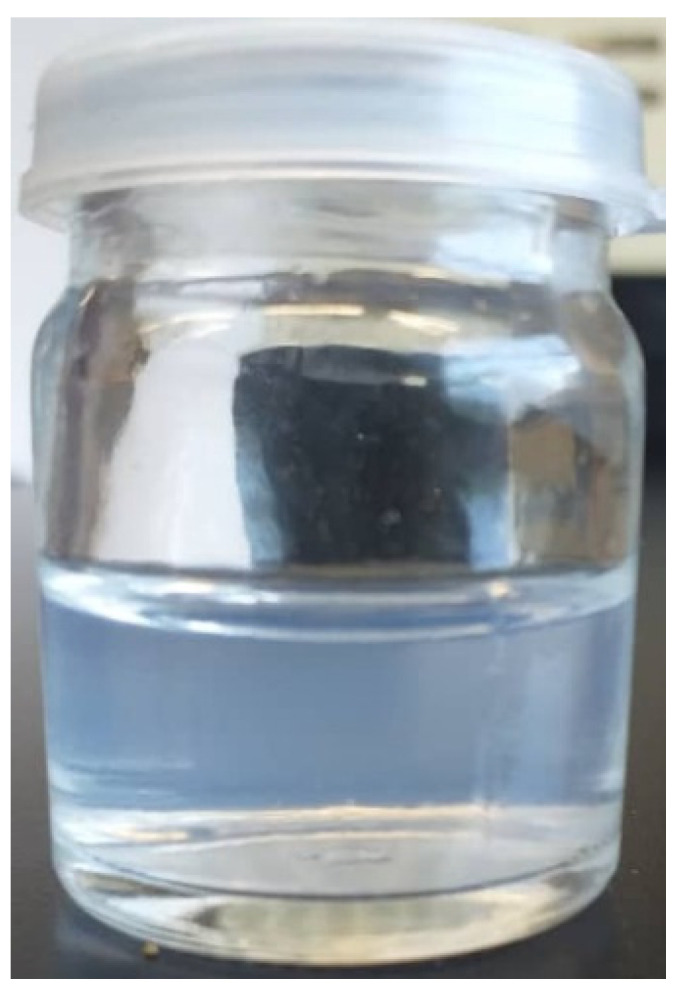
The view of plasticized composite electrolyte based on polyvinyl alcohol-potassium carbonate-silica (PVA-K_2_CO_3_-SiO_2_).

The synthesized CPEs, a typical one shown in Figure 1, were observed to be homogenous, clear, and viscous.

### 2.3. Physicochemical Characterization

Fourier transform infrared (FTIR) analysis was conducted on an FTIR spectrometer (Bruker Instruments, model Aquinox 55, Oberkochen, Germany) in the 4000–400 cm^−1^ range using KBr pellets with the scanning resolution of 4 cm^−1^. The morphology of the synthesized samples was examined using field-emission scanning electron microscopy (model: Zeiss Supra 55VP, Oberkochen, Germany) at 20 kV. Before the testing, the samples were vacuum dried for a period of 10 min on a carbon tape attached to an aluminum stub. The samples were coated with gold (Au) film in an evaporator to avoid charging and were then scanned at a magnification of 20 k and at an operation voltage of 30 kV [23].

The thermal stabilities of the as prepared CPEs were evaluated using thermogravimetric analyzer (TGA), model SHIMADZU DTG-60/60H (Kyoto, Japan). About 3 mg of the samples were heated at a heating rate of 10 °C/min in N_2_ atmosphere from room temperature to 900 °C and the weight of the sample was measured as a function of the temperature. Differential scanning calorimetry (DSC) (Model DSC Q2000 V24.11, Oberkochen, Germany) was used to examine thermal properties of the samples. The glass transition temperature (*T_g_*) of the samples were recorded at a heating scan rate of 10 °C/m in from 50 °C to 400 °C under N_2_ atmosphere. A sample weight of about 4 mg was used under nitrogen flow [7].

### 2.4. Electrochemical Characterization

#### 2.4.1. Electrochemical Impedance Spectroscopy (EIS)

**Figure 2:**
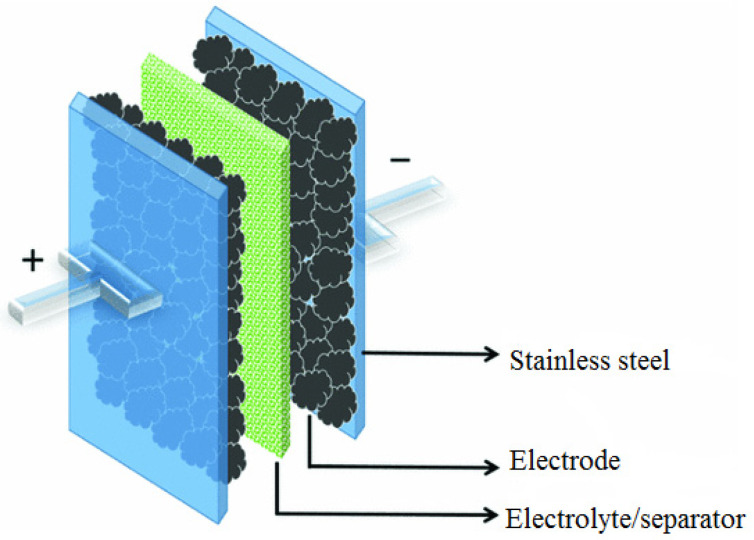
Schematic diagram showing 2-electrode system for electrochemical impedance spectroscopy (EIS) analysis.

Electrochemical impedance spectroscopy (EIS) analysis was conducted using AUTOLAB/AUT51018 (Metrohm Ltd, Herisau, Switzerland) (potential/galvanostat) in order to evaluate ionic conductivity of the developed CPEs. As shown in Figure 2, the system consisted of a separator containing the developed electrolytes placed between two stainless steel blocking electrodes where the testing was conducted within 0.1 to 10^5^ Hz frequency range at 303.15–383.15 K [24].

Ionic conductivity of the prepared CPEs was calculated using Equation (1) as described previously [25]:

(1) $$\sigma =\frac{t}{R_{b}A}$$

where *t* is the thickness of the samples (cm), *R_b_* (Ω) is the bulk resistance, and *A* is the electrode-electrolyte contact area (cm^2^).

#### 2.4.2. Linear Sweep Voltammetry (LSV)

Potential window or cell voltage of the prepared CPEs were studied using linear sweep voltammetry (LSV). The analysis was conducted at a scan rate of 10 mVs^−1^ in the potential range from −4 V to 4 V following the setup described in Figure 2 (AUTOLAB/AUT51018).

## 3. Results

### 3.1. Structural Analysis of CPE

**Scheme 1:**
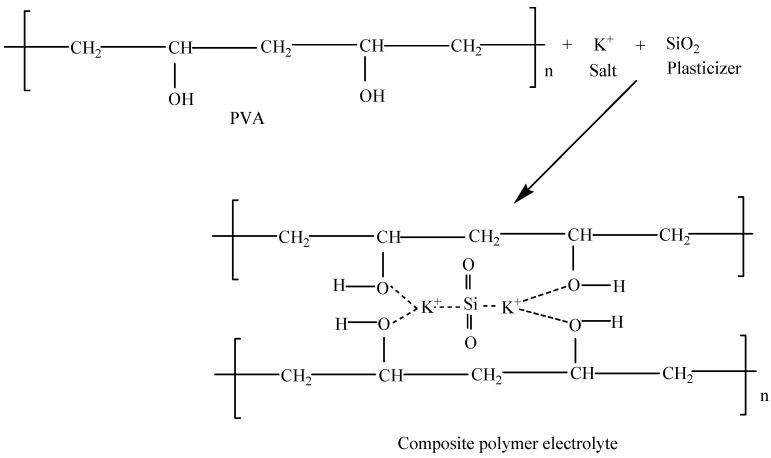
Possible structure for the synthesized PVA-K_2_CO_3_-SiO_2_ composite electrolyte.

The CPEs in this study were prepared by incorporating conducting salt (K_2_CO_3_) and the filler (SiO_2_) into the polymer host (PVA) as described in the experimental section. To elucidate the principle of ion conduction in CPEs, the mechanism of ion transport in polymer electrolytes was proposed as illustrated in the following scheme (Scheme 1). The K_2_CO_3_ can be easily dissociated into cations (K^+^) and anions (CO_3_^2−^) upon interaction with the solvent (dissolution) as shown in Equation (2).

(2) $$K_{2}{\mathrm{CO}}_{3(s)}⟶{K^{+}}_{\left(\mathrm{aq}\right)}+{{{\mathrm{CO}}_{3}}^{2-}}_{\left(\mathrm{aq}\right)}$$

The K^+^ chemically coordinates with the polar (−H) groups of PVA in the presence of SiO_2_. This coordination results in the formation of weak bonds between hydroxyl group, K^+^, and the SiO_2_ which results in complex formation as shown in Scheme 1 [26].

In addition, the partial or weak bond formed between K^+^ and SiO_2_ with the polar groups of the polymer could aid in hastening the conduction of ions since the ions are in a moveable state [26]. Similarly, silica material was found to affect the changes in the morphological structure of the electrolytes [20]. The silica external surface may chemically coordinate with the ions and subsequently deliver extra site generating favorable tunnels or ways for ion transport within the locality of the electrolytes [26]. The chemical coordination within the electrolytes might increase the conduction sites for the movement of ions, thus creating extra conducting ways for migration of ions [21,27]. Previous studies have reported that the silica particles incorporated into the polymer matrix could be blended with the electrolyte material and the conductivity can be improved. This can be credited to its greater volume division of the amorphous phase [28,29].

The linkage between the polymer polar groups, the cations (K^+^), and the silica material creates a large amount of ion conducting site and a well improved interfacial coordination, accordingly resulting in improvement in ionic conductivity of the electrolyte [30]. The interactions between metal ions from the salts and the plasticizing effect from the silica with hydroxyl groups (−OH) of the polymer result generally from electrostatic attractions and hence lead to the coordination bond formation. There are features that generally affect salt-polymer-filler coordinations, such as type of hydroxyl group bonded to the backbone of the polymer, region among the functional groups, molecular weight, compositions, nature and charge of metal cation, counter ions, and the degree of branching [31]. The cations from the salts move from one interaction site when an electric field was subjected to the system owing to the effect from the silica [32]. Previous studies reported that ions from the salt and the plasticizer that are connected with the hydroxyl groups transfer through re-coordination along the polymer backbone. Therefore, the chains of the polymer are doubled over to form channels, in which the ions (mostly cations) are coordinated and situated by the OH group of the polymer. These channels generate passages, providing ways for the ions’ movement in the system [33].

Moreover, it was reported that addition of filler into the CPEs was found to decrease the number of active centers, and consequently weaken molecular forces between the polymer chains [32]. Hence, this leads to a decrease in the inflexibility of the host polymer and consequently disturbs the thermo-mechanical properties of the polymer [30,31]. Moreover, addition of filler was found to affect glass transition temperature (*T_g_*) of the polymer system [30]. Thus, this leads to the decrease in crystalline region of the composites and subsequently increases ion dissociations where a significant enhancement in charge carriers transport is achieved that leads to higher ionic conductivity. The formation of complex between polymer, salt, and the filler (PVA-K_2_CO_3_-SiO_2_) will lead to an increase in entropy, which results in the segmental motion being increased. The increase in segmental motion was reported to decrease crystallinity and improve flexibility of the electrolyte material [33]. The incorporation of silica particles into polymer backbone can increase the ion dissociation to yield more free cations for conductivity [27,34].

### 3.2. Physicochemical Characterization

#### 3.2.1. Chemical Structure

**Figure 3:**
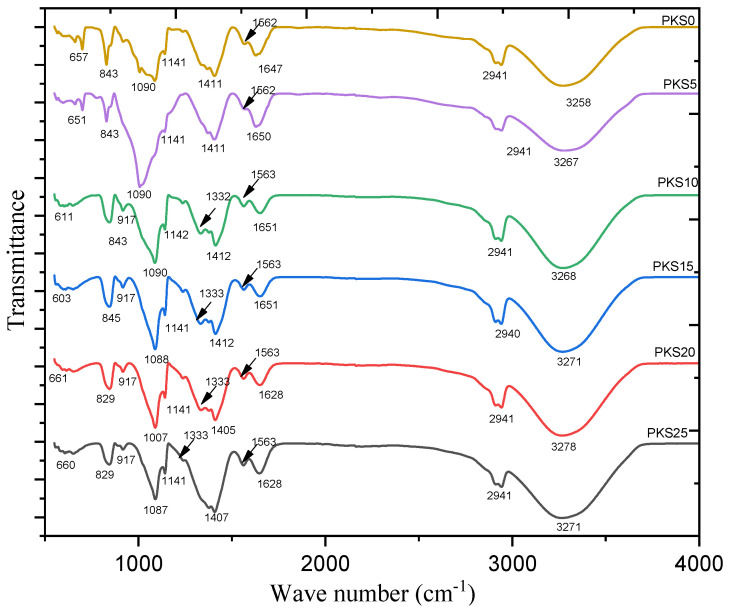
FTIR spectrum of PVA-K_2_CO_3_ (PKS0), PKS5, PKS10, PKS15, PKS20, and PKS25 composite polymer electrolytes.

To affirm the interaction and coordination between salt, polymer, and the filler (silica) in CPEs, the FTIR spectra of the samples were analyzed and the result is presented in Figure 3 and Table 2. The absorption peak of the CPEs shows various peaks between 4000 cm^−1^ and 400 cm^−1^. The absorption spectrum at 3258 cm^−1^ band for the PVA/K_2_CO_3_ with no SiO_2_ (PKS0) is attributed to the stretching of O–H vibrations of alcohol group [27,35]. The broad peak appearing at 2941 cm^−1^ is associated with the asymmetric C−H stretching. The sharp peak located at 1647 cm^−1^ is assigned to the C=O stretching of carboxyl group [36]. The appearance of peaks in the region of 1562 cm^−1^ could be assigned to the strong C=C stretching vibration from α, β-unsaturated ketone of the polymer composites [37]. Furthermore, the peak at 1411 cm^−1^ belongs to bending vibrations of CH_2_ while the band 1141 cm^−1^ occurred because of the CH twitching vibrations. The bands at 1090 cm^−1^ belong to the C–O broadening of the acetyl group present in the PVA structure while the absorption spectrum at 843 cm^−1^ has been allocated to C–H rocking vibrations. Finally, the small peak at 657 cm^−1^ is assigned to the free CO_3_^2^^−^ anions, which indicates the mobility of the solvated K^+^ and CO_3_^2^^−^ ions in the CPE system [38].

FTIR plots for samples PKS5 up to PKS25 depict the spectra of as prepared CPEs with 5, 10, 15, 20, and 25 wt.% of SiO_2_, respectively. It was observed that the peaks of O–H widening, asymmetric C−H widening, C=O enlarging, C=C broadening, and all other peaks were still found with few changes and little shifting in the peak intensities [7]. These changes and shifting in the peak intensities are reported to be important in order to establish the complex formation between the host polymer (PVA), salt (K_2_CO_3_), and the filler (SiO_2_). For instance, the shifting of hydroxyl band of the composite polymer electrolyte (PKS) from 3258 to 3271 cm^−1^ when blended with SiO_2_ and the formation of new spectrum at 917 cm^−1^, and 1333 cm^−1^ corresponding to symmetric and asymmetric Si–O–Si expanse, respectively, indicate the polymer-salt-silica complexation [39,40]. It was also observed from the spectra of the PKS15 that the peak of CH wagging at 843 cm^−1^ moved to higher wavenumbers (845 cm^−1^) than the other electrolytes, which designates shortened bond lengths of gel-like structures in the electrolytes [35,41]. These indicate that SiO_2_ interacts with PVA-K_2_CO_3_ in the form of a three-dimensional polymeric network (Scheme 1). In other words, SiO_2_ and PVA-K_2_CO_3_ chains exist in the form of CPEs [22].

#### 3.2.2. Miscibility and SiO_2_ Presence

**Figure 4:**
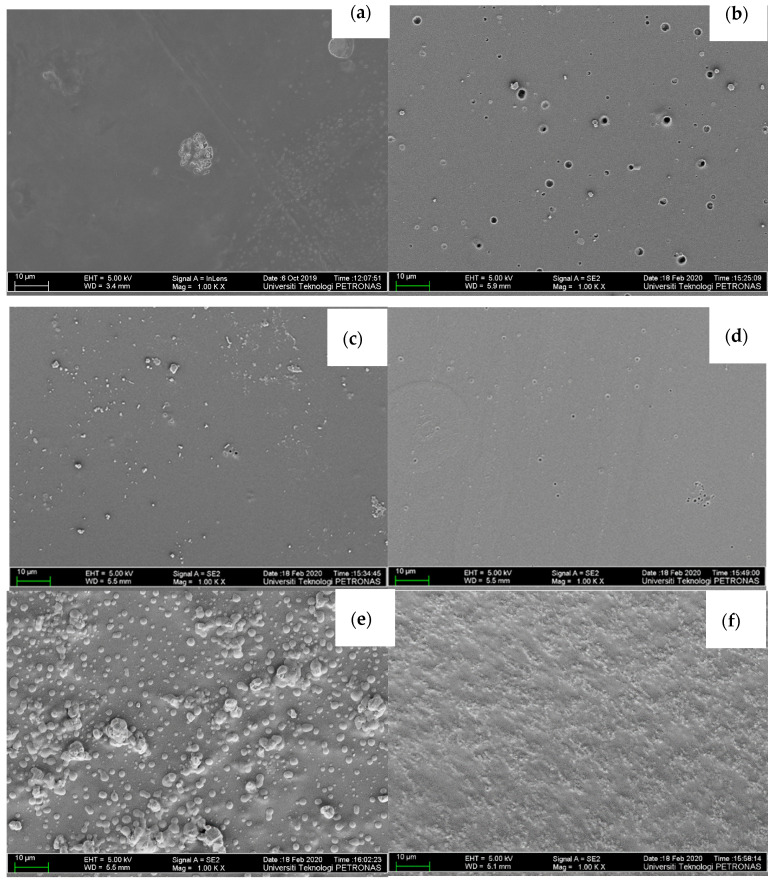
FESEM micrographs for the surface of (**a**) PKS0, (**b**) PKS5, (**c**) PKS10, (**d**) PKS15, (**e**) PKS20, and (**f**) PKS25 composite polymer electrolyte.

The surface morphology of the PVA-K_2_CO_3_ (PKS0) and the composite (PVA-K_2_CO_3_-SiO_2_) electrolytes were studied using FESEM and the results are presented in Figure 4a–f. Figure 4a shows the surface morphology of the non-filler or filler-free electrolyte (PKS0) and it was observed that the sample demonstrated a smooth and homogeneous surface showing great miscibility between salt (K_2_CO_3_) and the polymer. Figure 4b–e shows some agglomeration between PVA, K_2_CO_3_, and SiO_2_ proving successful accumulation of silica in the inter-spherulitic region (amorphous region) which further improved with an increase in amorphosity of the polymer due to incorporation of filler [40,42]. With the dispersion and incorporation of 5–15 wt.% SiO_2_, the CPEs showed uniform and smooth surfaces with white granules and the formation of more porous structures, indicating better compatibility between host polymer, salt, and the filler as can be observed in Figure 4b–d. It was also observed that incorporation of 5–15 wt.% SiO_2_ particles changed the microstructure of the composites into a more amorphous structure and with development of some voids that serve as ion migration paths. The smooth surface of the electrolytes indicates the homogeneous dispersion of the SiO_2_ particles. This was possible due to formation of weak bonds between the hydroxyl group of the polymer (−OH), K^+^, and the SiO_2_ [21]. As can be observed, SiO_2_ is presented as white granules in the PVA-K_2_CO_3_ composites confirming successful dispersion of the filler and the formation of PVA-K_2_CO_3_-SiO_2_ composites. Moreover, addition of 5–15 wt.% of filler gave a positive change to the microstructure of the electrolyte and this could be ascribed to the good coordination and adhesion between the surface matrix of the polymer and SiO_2_ owing to the good homogeneous dispersion [42]. The increase in amorphous nature of the composites is highly favorable for attaining strong interaction between electrolytes and other components in the systems. This structure would support an improvement in ionic conductivity of composite electrolytes [41].

However, the surface became coarser with the addition of more than 15 wt.% (Figure 4e,f). Similarly, a substantial growth in the concentration of white granules was also observed, which could be attributed to recrystallization (formation of more crystalline region due to the high content of the silica) and aggregation of the filler in the polymer, since the polymer host is unable to accommodate large amounts of the SiO_2_ [42]. A previous study reported that large amount of filler may cause an uneven dispersion and agglomeration on the polymer matrix surface owing to the high surface energy and poor adhesion between polymer and the filler [40]. Hence, more agglomerations were observed in the composites with more than 15 wt.% SiO_2_ which may lead to the loss of a significant amount of charge carriers that would subsequently lead to a decrease in conductivity [10,42].

**Figure 5:**
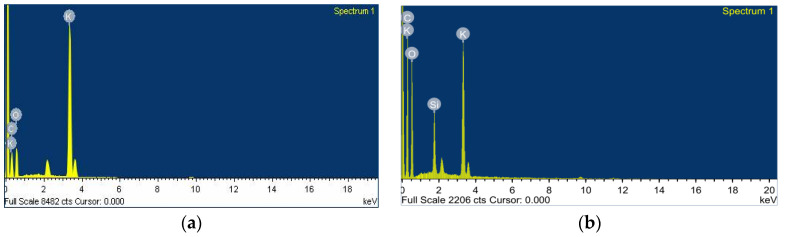
Energy-dispersive x-ray (EDX) plots representative of the composite polymer electrolyte (**a**) PKS0 and (**b**) PKS15, showing successful incorporation of Si into the PVA-K_2_CO_3._

Correspondingly, Figure 5 presents results of energy-dispersive x-ray analysis (EDXA) analysis; it was observed that Si was successfully incorporated into the surface of the PVA-K_2_CO_3_ composites. This is anticipated to increase the amorphous region of the composites electrolytes that subsequently improved ionic conductivity of the CPEs.

#### 3.2.3. Thermal Stability

**Figure 6:**
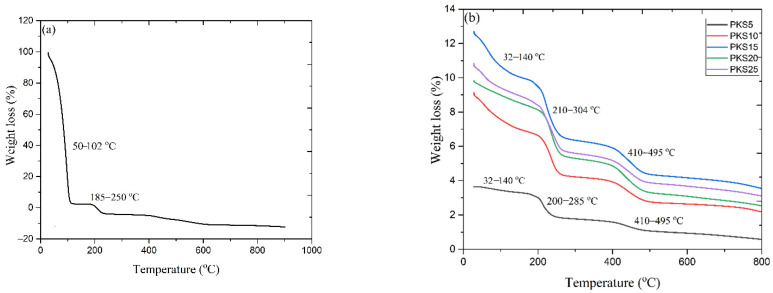
TGA thermograms of (**a**) PKS0 and (**b**) PKS5, PKS10, PKS15, PKS20, and PKS25 composite polymer electrolyte.

Thermal stability is the most significant variables deciding stability and suitability of polymer electrolytes in every energy storage device [40]. Figure 6a,b portrays thermogravimetric analysis for filler-free electrolytes (PKS0) and electrolyte with fillers. The filler-free electrolytes show two major weight losses, one located around 50−102 °C that could be ascribed to the removal of water molecules owing to the hydroscopic nature of the polymer and impurities bonded to the polymer electrolytes. Another minor weight loss located at 102−185 °C is attributed to the elimination of side-groups, chain scission, and the cessation of the PVA backbone at higher temperature as well as strong effect of the K_2_CO_3_ [29].

**Scheme 2:**
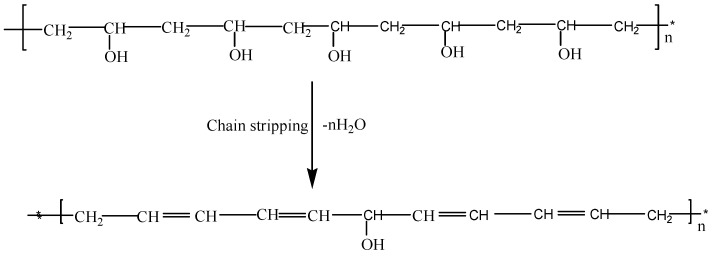
Polymer chain stripping mechanism.

However, three thermal degradation steps were noticed for all samples with fillers. The first degradation was noticed around 32−140 °C because of the removal of moisture attached to the composites as described previously. The minor weight loss around this phase can be associated with the exclusion of unwanted objects in the samples [27]. The second step occurred between 210 and 304 °C while the third step followed between 410 and 495 °C. It has been confirmed that, the breakdown of polymer electrolytes especially PVA starts with chain ‘‘stripping’’, which might result in ether cross-linkage formation i.e., removal of water molecules together with the conjugated double bonds formation, generating polyene structures at temperature above 200 °C as shown in Scheme 2 [27,29,38].

Upon addition of filler, the mass loss and the thermal decomposition were observed to increase significantly due to the presence of silica in the PVA matrix. This indicates the influence of the complex formation between the polymer, filler, and the salt [21]. The weight loss of samples with filler was observed to be greater than filler-free samples (210 and 304 °C), which suggests that the highly conducting electrolytes display better thermal properties [19,29]. The final weight loss (410 and 495 °C) is linked to the cessation of the PVA backbone [43]. At this temperature, the double bond of polyene structures shown in Scheme 2 would be broken down, and converted into low-molecular weight single bond aliphatic polymer products. Further than this phase, the polymer electrolytes’ weight remains unaffected [29]. Based on TGA analyses, thus, it can be established that the incorporation of silica material into PVA matrix successfully improved the general stability of the polymer electrolyte based on PVA-K_2_CO_3_-SiO_2_ composites.

#### 3.2.4. Glass Transition Temperature

**Figure 7:**
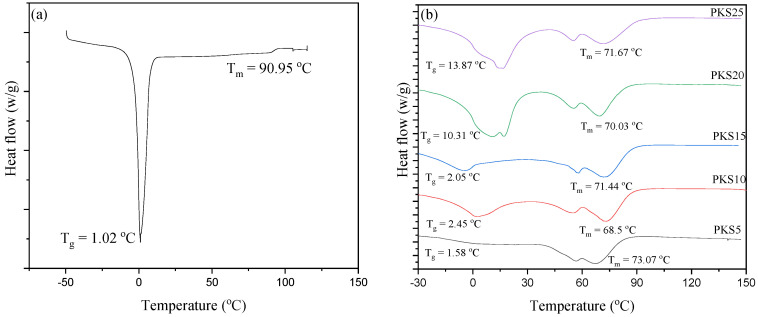
DSC scans for (**a**) PKS0 and (**b**) PKS5, PKS10, PKS15, PKS20 and PKS25 composite polymer electrolyte.

To further study the effect of silica filler on the polymer-salt electrolytes, DSC analysis was conducted and recorded for different polymer electrolytes and the results are presented in Figure 7a,b. For PKS0, an endothermic peak at 90.95 °C linked to the melting point (*T_m_*) of the polymer-salt electrolytes was observed. However, a decrease in the *T_m_* (*T_m_* = 73–68 °C) of the electrolytes containing silica material was observed, indicating an increase in amorphous structure of the samples which is due to the incorporated SiO_2_ particles, as earlier discussed in FESEM results (Figure 4). This increase in amorphosity is associated with the good coordination between PVA and filler that decreased the extent of crystalline region of PVA into the amorphous region and subsequently improved the conductivity. The decrease in *T_m_* indicates that the transition from crystalline to amorphous phase has occurred, which has a vital role in the movement of ions. Therefore, an improvement in ionic conductivity is expected with the increase in SiO_2_ composition [10]. A similar study of decrease in *T_m_* was reported previously while incorporating silica into a polymer [22].

Conversely, addition of SiO_2_ was found to increase *T_g_* of the electrolytes as can be seen in Figure 7. It was reported that an increase in *T_g_* can affect polymer segmental motion and hence ionic conductivity. The values of *T_g_* of the entire electrolyte with fillers (PKS5–PKS25) were observed to increase with the addition of the SiO_2_ showing the successful formation of composites. A previous study reported that the *T_g_* of polymer materials increases upon addition of plasticizers or fillers due to the partial coordination bonding during complex formation [44]. The increase of *T_g_* observed in this study indicates the effect of the SiO_2_ that relaxes the PVA backbone and hence yields bendable backbone that aids in stimulating easy migration of ions in the electrolyte systems [45]. The addition of plasticizer or filler into the polymer materials was reported to enhance ionic movement because of the growing ionic coordination in the host polymer [46]. Low *T_g_* could be attributed to the reduction in crystallinity due to the incorporation of the silica material. Polymers with low *T_g_* are desirable for delivering better plasticity of the PVA chains to provide faster ionic movement. This ionic movement in the systems of polymer matrix could be associated with the confined fundamental relaxations that are categorized by the *T_g_* of the polymer [29,37,47].

#### 3.2.5. Crystal Phase

**Figure 8:**
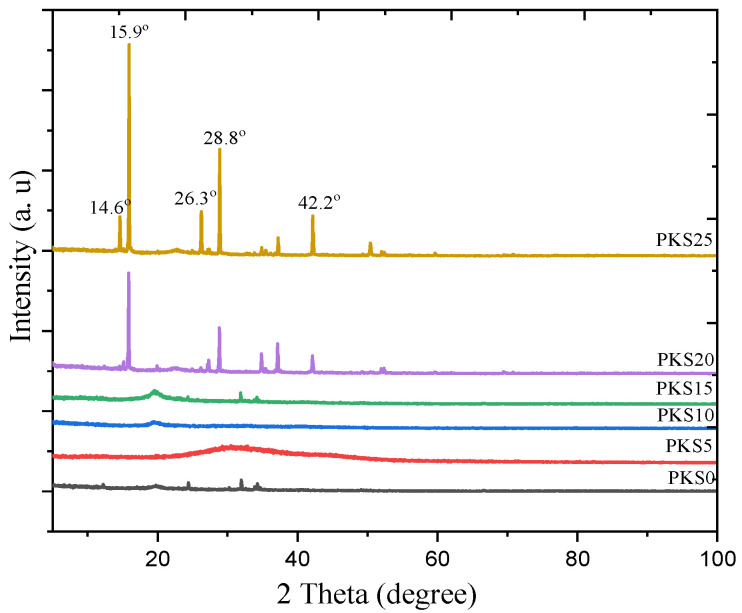
The XRD patterns of PKS0, PKS5, PKS10, PKS15, PKS20, and PKS25 for PVA-K_2_CO_3_-SiO_2_ composites.

XRD analysis allowed detection of crystalline or amorphous phases of the electrolyte before and after incorporation of SiO_2_ into the parent PVA-K_2_CO_3_. The XRD patterns of PVA-K_2_CO_3_ (PKS0) and PVA-K_2_CO_3_-SiO_2_ are shown in Figure 8. It is clear that the natural framework structure of PVA-K_2_CO_3_ was conserved after incorporation of lower wt.% (5–15 wt.%) of SiO_2_ which could be attributed to the good dispersion of SiO_2_ into the PVA-K_2_CO_3_ matrix and good miscibility between PVA, K_2_CO_3_, and SiO_2_ which leads to the successful PVA-K_2_CO_3_-SiO_2_ composite formation. As can be observed, PKS5 shows broad and distinct characteristic structure, and this could be partly attributed to the initial dispersion and incorporation of the filler into the surface of the polymer matrix and to the weak crosslinking reactions between the polymer matrix and the filler due to the low amount of the SiO_2_ content [17]. The results revealed that the incorporation of moderate amounts of filler (5–15 wt.%) had a significant effect on the electrolyte and did not cause any serious change in morphology of the PVA-K_2_CO_3_, and the amorphous structure of the electrolyte was almost completely preserved which may result in significant increase in conductivity of the PVA-K_2_CO_3_-SiO_2_ electrolyte. It was reported that the amorphous characteristics of the composite electrolyte after successful incorporation of filler was found to accelerate ionic transport in the polymer framework, which further enhanced the ionic conductivity of the CPE [11,27].

However, new peaks appeared after incorporation of higher weight percentages of SiO_2_ (20–25 wt.%) with characteristic diffraction pattern of chabazite phase showing Si peaks at 2θ = 14.6°, 15.9°, 26.3°, 28.8°, and 42.2° corresponding to (1 0 1), (1 1 0), (0 2 1), (2 2 0), and (202) planes, respectively. This confirmed the successful dispersions of SiO_2_ into the parent PVA-K_2_CO_3_. Consequently, a substantial increase in crystallinity was observed which could be attributed to the recrystallization and aggregation of the filler in the PVA-K_2_CO_3_ since the polymer host is unable to accommodate large amount of the filler [42].

### 3.3. Electrochemical Properties

#### 3.3.1. Resistance

**Figure 9:**
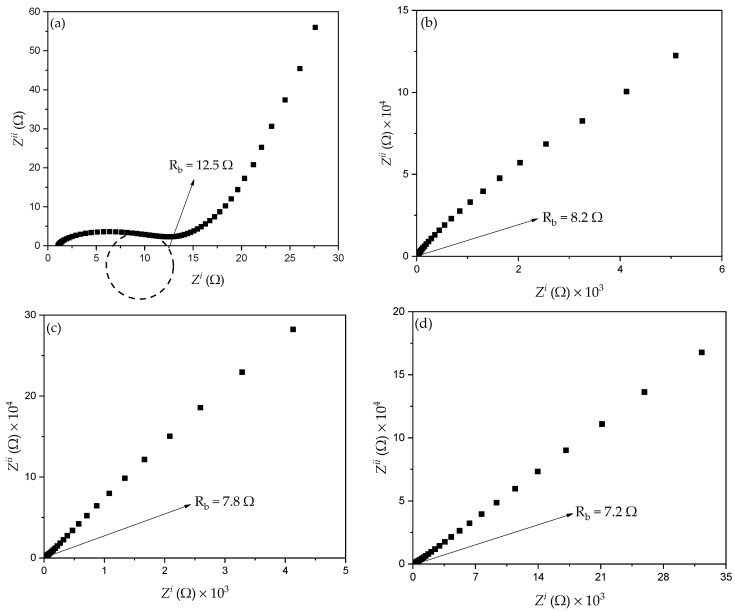

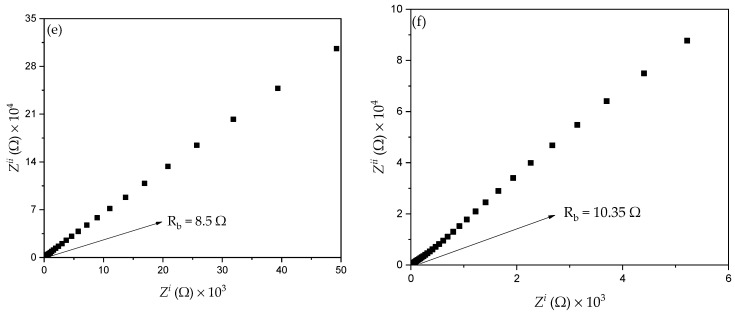
Cole–Cole plots for (**a**) PKS0, (**b**) PKS5, (**c**) PKS10, (**d**) PKS15, (**e**) PKS20, and (**f**) PKS25 composite polymer electrolytes at 373.15 K.

A previous study reported that the conductivity of CPEs depends mainly on ionic mobility and the concentration of ion carriers [10,48]. Thus, the EIS technique was used to evaluate ionic conductivity and ionic conductors in the polymer electrolytes and to understand the electrode–electrolyte interface behavior. Figure 9a,b shows the results of the EIS analysis for all samples with different amount of filler at 373.15 K. It can observed that the distinctive Cole-Cole plot of the PKS0 (Figure 9a) consists of a broadened semicircle at a higher frequency region with tilted spike, suggesting that the electrolyte is partly resistive and capacitive in nature [48]. The semicircle at the high frequency region is associated with the parallel combination of a resistor and a capacitor and a low frequency spike signifies formation of double layer capacitance at the electrode–electrolyte interface owing to ion migration at low frequency [49].

However, with the successful incorporation of filler, the semicircle of the un-plasticized sample disappeared which could be attributed to the increase in amorphous region of the polymer matrix that led to a decrease in (bulk) resistance. Hence, the Cole-Cole plots of the CPEs containing filler (Figure 9b–f) consist of a tilted spike at both high- and low-frequency regions which indicates that only the resistive component of the polymer material presents in the sample [25]. Similarly, in the EIS result, the capacitor must display a tilted spike when it is taken to be perfect [10]. It was reported that the long tails with no circle observed in the impedance plots denote the role of the ions or charge carrier dispersion and the effect of blocking electrodes (electrode polarization), hence their buildup at the interface of the electrode and electrolyte [49]. This increase of charge carriers at the interface of the electrode and electrolyte results in electrical double layer capacitances. The low and high frequency spike signifies double layer capacitance formation at the interface of electrode and electrolyte which could be associated with the movement of charge carriers at lower frequency [11]. Likewise, the linearly rising spike in the EIS plot reveals the high absorption of ions and thus specifies the capacitive nature of the system [50].

From the plots in Figure 9, the resistance at the high frequency region is obtained from the intercepts on the *x*-axis of the complex EIS plots. This resistance is known as the bulk resistance (*R_b_*) [27,51]. The *x*-axis intercept from the high frequency reveals the bulk properties of the electrolytes [47]. *R_b_* decreased with the increase in filler from 5 to 15 wt.% owing to the increase in carrier density and ion mobility *µ_i_*. It was also observed that the successful incorporation of SiO_2_ into PVA helped salt (ion) dissociation, thus decreasing the formation of ion aggregates. It was found that *R_b_* of PKS15 was lower when compared with *R_b_* of other samples. This could be associated with the fact that PKS15 appears to be more amorphous as observed in FESEM and DSC results (Figure 4 and Figure 7). Furthermore, high effect of SiO_2_ particles was a significant factor in reducing *R_b_* of the polymer material. This indicates the actual effect of SiO_2_ in increasing the interfacial contact at the electrode and electrolyte system. The movement and diffusion of ions or charge carriers in the electrode-electrolyte system might similarly be improved with low *R_b_*, causing an increase in ionic conductivity [41].

However, an increase in *R_b_* with increase in SiO_2_ particles beyond 15 wt.% was observed and this can be associated with the high *T_g_* (Figure 7) value of the samples that led to an increase in crystallinity of the polymer material. When high amount of SiO_2_ particles were incorporated into the polymer matrix, more crystalline phases were introduced to the electrolyte system, consequently leading to the increase in *R_b_*. It is very obvious that the crystalline phase in polymer matrix blocks the ionic motion and consequently causes a decrease in conductivity [10]. This is in agreement with the FESEM results discussed in Figure 4e,f where a substantial increase in the concentration of white granules was detected which could be attributed to the recrystallization and aggregation of the polymer electrolyte.

Further, an equivalent circuit of the EIS plots was proposed as shown in the inset of Figure 9a in order to provide information about interface properties of the electrolytes. The high frequency semicircle region of the EIS plots was made by a parallel combination of a capacitor and resistor, as denoted in the equivalent circuit model. The bulk properties (R_b_) of the electrolytes denote the capacitance and interfacial contact between the electrodes [2]. The capacitor represented by the capacitance of double layer (C_dl_) marks the formation of electrical double layer owing to the increase in charge carrier concentration at the electrode-electrolyte interface due to the diffusion of ions into the porous surface of the electrode and consequently provide high ionic conductivity. The C_dl_ was joined in parallel with charge transfer resistance (R_ct_) and a capacitance that were incorporated in series in order to evaluate the contribution of pseudocapacitance [10]. Thus, the results established that the successful incorporation of K_2_CO_3_ and SiO_2_ into the polymer increase ion diffusion in the electrolyte system and therefore support adsorption of ions at the electrode-electrolyte interface, which leads to the increase in ionic conductivity [23]. The resistance R_ct_ signifies charge transfer resistance of the CPEs on the charge absorption across the electrode/electrolyte interfaces. Hence, the ions are essential to overcome the resistance in order to form the electrical double layer. The total internal resistance of the cell, which is the combination of R_b_ and R_ct_ was computed from the intercept of the semicircle [2].

#### 3.3.2. Ionic Conductivity

**Figure 10:**
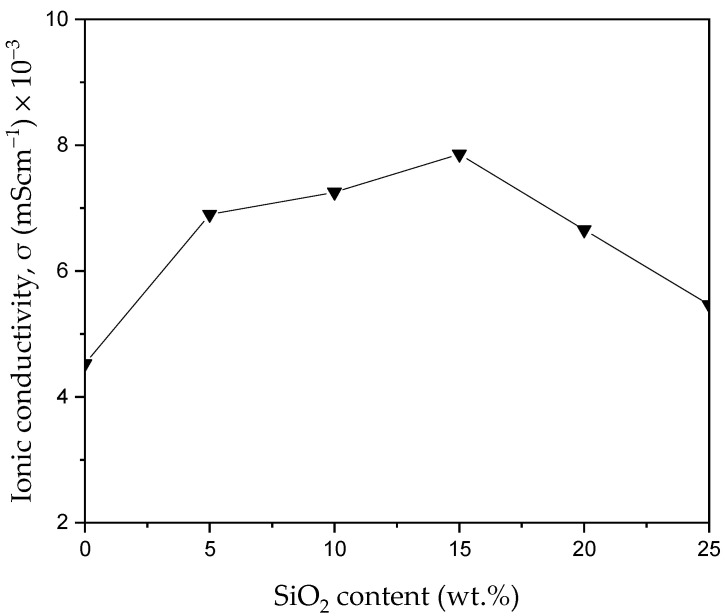
Plots of conductivity of the PVA-salt-SiO_2_ CPE with different SiO_2_ contents at 373.15 K.

To study the effect of filler on ionic conductivities based on PVA-K_2_CO_3_ composites, the ionic conductivities were measured as a function of silica content at 373.15 K. Figure 10 depicts the ionic conductivity of the synthesized samples at different wt.% silica content calculated using impedance results by applying Equation (1). As can be observed, with the inclusion of the silica particles, the conductivity improved. This conductivity continued to slowly increase with the incorporation of 5–15 wt.% of silica particles (PKS15). The highest conductivity of 7.86 × 10^−3^ mScm^−1^ was achieved at 15 wt.% of SiO_2_ as compared to conductivity of the non-filler electrolytes (PKS0) of 4.50 × 10^−3^ mScm^−1^. The improvement in conductivity can be ascribed to the influence of the SiO_2_ [29,52]. Strong influence of SiO_2_ helps to temper with the PVA-salt complexation and thus enhanced the plasticity of the PVA chain. The influence of the SiO_2_ aids ions to be easily migrated in the PVA chain with very flexible chains polymer [21,53].

Moreover, SiO_2_ enhanced the amorphous region of the polymer, which results in weakening the temporary coordinative bonds between the molecules in the crystalline phase and transforming the polymer into flexible complexes [39]. Likewise, filler encourages the conduction process through provision of new conducting sites. This could be achieved by coordinating the cations from the salt with the polar groups of the side chain in the backbone of polymer [53]. This coordination not only offers more sites for the conduction process, but also offers more ions in the composites. This also results in additional free charge carriers [25]. Moreover, with the successful dispersion of silica particles into the polymer-salt complex, an active ionic tunnel for the conduction process was created with the double layer formation. This led to enhancing the electrical potential between the electrodes when the electric field is applied. This results in the assumption that the charge carriers are mobile ions as discussed in EIS analysis [22]. Consequently, the highest conductivity was achieved in the PKS15 electrolyte. This type of effect by the filler was also reported earlier, where the complexations between the polymer and the ions were affected by the silica material [53]. As suggested earlier, the SiO_2_ particles as a filler can also act as a “solvent” to stimulate dissociation of ions that subsequently increase the ionic conductivity [54].

However, when the amount of filler exceeds 15 wt.%, the conductivity starts to decline. This shows that the greater amount of filler did not contribute to the increase in conductivity. High amounts of SiO_2_ resulted in the re-crystallization process (seen in FESEM result, Figure 4e,f) in the composites which caused the PVA chains to become inflexible and thus significantly reduced the movement of ions [21]. Similarly, the decrease in ionic conductivity at higher amounts of filler can be ascribed to the agglomeration of charge carriers in the polymer electrolytes. Excessive ions in the polymer matrix were reported to cause the formation of aggregates and ion pairs. These aggregates might cause the blocking of the ions tunnels (ways) and deter the migration of ions in the systems, which leads to a decrease in conductivity. In addition, the number of ions available for conduction is decreased in the presence of ion aggregates. At a high amount of filler, the cations (K^+^) from the salt could relatively form pairs with anions instead of providing carrier charges for ion movement [17,29]. These cations form neutrals with anions from the hydroxyl group of the polymer and hence transform the cations into an immovable state. Consequently, the conducting sites of the polymer electrolytes could be reduced and eventually decrease the conductivity. These aggregates and neutral pairs obstruct the conducting ways and decrease the flexibility of polymer [55,56]. Similarly, an increase in *T_g_* is related to the increase in the crystalline nature of the polymer. Thus, conductivity decreases with the increase in *T_g_*. As can be observed samples with highest *T_g_* values (PKS20 and PKS25) exhibited lowest conductivity since conductivity is higher when the electrolytes are in amorphous form.

The values of ionic conductivity achieved in this study were higher with more than two orders of magnitudes than the values reported earlier using different plasticizers/fillers [57,58]. Table 3 compares the results obtained in this study with previous studies earlier reported. It can be observed that the prepared electrolyte in this study produced higher ionic conductivity at ambient temperature than other electrolytes reported using different plasticizers/fillers such as MoO_3_ [57], KI [58], NH_4_I [59], and NH_4_Br [60]. The higher performances displayed by the prepared electrolyte in this study could be due to the strong effect of silica particles that helps ions to be easily migrated in the PVA chain with very flexible chains polymer. Similarly, the ability of the prepared electrolyte to stick and penetrate into the porous structure of the fiber (separator) due to the excellent penetrating and sticking ability of the gel electrolyte could be another reason for achieving higher conductivity and potential window.

#### 3.3.3. Temperature-Conductivity Relationship

**Figure 11:**
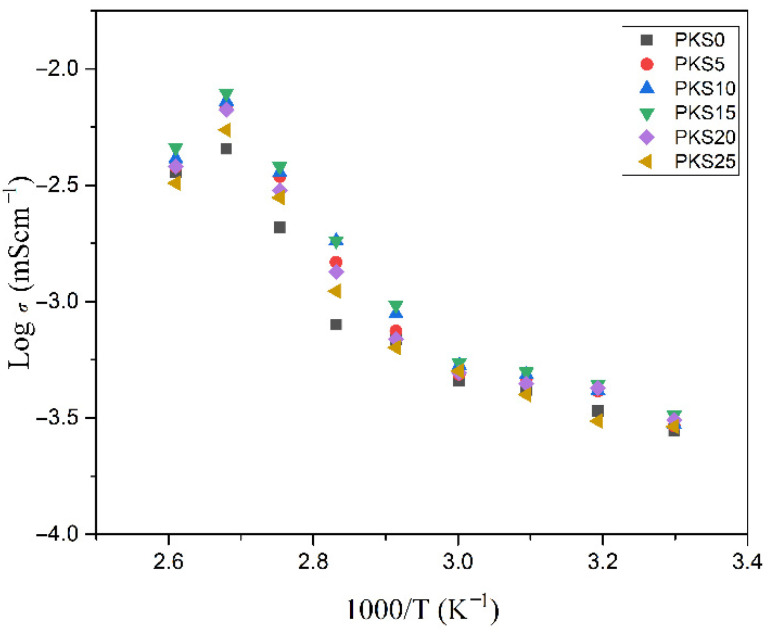
Conductivity versus temperature of PVA-K_2_CO_3_–SiO_2_ CPEs.

Figure 11 portrays ionic conductivity which is the most important parameter for polymer electrolytes against 1000/T of the prepared polymer electrolytes in the temperatures between 303.15–383.15 K. The results show that the ionic conductivity slowly increased with the increase in temperature for all the prepared electrolytes. This increase in conductivity with increase in temperatures could be attributed to the transformation of PVA from semi-crystalline to an amorphous phase. Previous reports similarly linked the increase in conductivity with the temperature increase to the transformation of polymer matrix from semi-crystalline to an amorphous nature [64]. It was reported that formation of the amorphous phase in the polymer electrolytes helps ease migration of ions or charge carriers. This is because as the temperature increase, an amorphous phase of the PVA slowly increases, which helps polymer chain acquire rapid internal vibrations whereby a segmental motion is created owing to the bond spins. This successfully helps in ionic migrations and consequently results in an enhancement in ionic conductivity [65].

It can be observed that the prepared electrolyte in this study exhibits mixed behaviors of Arrhenius and Vogel–Tammann–Fulcher (VTF) models. For instance, the linear increment in conductivity with the rise in temperature from 303.15 to 333.15 K suggests that migration of charge carriers or ions occurs over the temperature-activated routes and hence follows Arrhenius trend [64]. As shown in Figure 11, ionic conductivity increased linearly with the rise in temperature from 303.15 K until 333.15 K before it began to form a curve at higher temperatures (above 333.15 K). This could be attributed to the enlargement of polymer matrix with the increase in temperature, which creates an empty space or free volume where ionic movement occurs. This subsequently enhances the movement of charge carriers or ions and lessens the ion cloud influence at the electrode and electrolyte system. Therefore, the linear relationships noticed in all the electrolytes at the stated temperatures indicate that the temperature dependency of ionic conductivity in the temperature range considered is of Arrhenius type given by Equation (3) [31]:

(3) $$\sigma =\sigma_{o}\exp\left(\frac{-E_{a}}{k_{B}T}\right)$$

where *σ_o_* is a pre-exponential factor, *E_a_* is the activation energy, *k_B_* is the Boltzmann constant, and *T* is the temperature (K).

Previous studies reported that rise in temperature may affect fast vibration modes of the molecules, which stimulates the rotation of bonds in the polymer [10]. Moreover, the ionic association within the polymer segmental motion could be easily separated from the weak bonds with the hydroxyl groups at higher temperature. Hence, ionic motion improved with increase in temperature. The amplitude of the mode of vibrations was found to improve with the increase in temperature and consequently, the thermal vibration increases the decoupling level of charge carriers [28]. More ions could migrate in the polymer matrix with greater decoupling frequency as the number of mobile ions that is responsible for ionic movement increases and hence more ionic conduction would be generated resulting in higher ionic conductivity. In addition, the polymer matrix could be expanded because of the higher amplitude of vibration at higher temperatures. Hence, this generates more space for the conduction process, which leads to ions decoupling and easily migrating, and results in ionic conductivity increase [10,53]. 

Consequently, at temperatures above 333.15 K, all the electrolytes exhibited a VTF model behavior by forming a curvature. Here, to have better understanding into the temperature dependence of the conductivity, σ above 333.15 K, the conductivity data were fitted to the VTF equation. The VTF equation, which has been used to describe various dynamical processes in glassy and polymeric systems, is expressed as in Equation (4) [31]:

(4) $$\sigma \left(T\right)=\;AT^{-1/2}\exp[-\frac{B}{k_{B}\left(T-T_{0}\right)}]$$

where *A* is a pre-exponential factor, *k_B_* is the Boltzmann constant, *B* is the pseudo-activation energy, *T*_0_ (*T*_0_ *= T_g_* − 50 K) is the absolute temperature, and *T*_g_ is the glass transition temperature. Previous studies attributed the non-linear behavior of conductivity to the polymer segmental motion, which assisted in mobility of ions. Similarly, it has been reported that the curvature or non-linearity behavior of ionic conductivity versus temperature of polymer electrolytes could be attributed to the fact that the ion transport model is assisted by the polymer segmental motion [29,64]. Based on the VTF model, the curving behavior of the Arrhenius plots with the rise in temperature could be ascribed to the existence of a strong inter-relation between the ionic motion and polymer segmental relaxation. This also implies that the polymer segmental motion and ionic motion are well coupled with each other. Therefore, it is clear from this finding on Arrhenius and VTF models that the increase in ionic conductivity with the increase in temperature could be associated with the increment in both segmental mobility and hopping rate [9]. Moreover, dielectric constant and ion dissociation energy was reported to have a great influence on the conductivity behavior of a polymer electrolyte [31].

However, the conductivity of all the synthesized electrolytes was observed to decrease at temperatures greater than 373.15 K. This decrease can be ascribed to the generation of grain boundaries within the polymer matrix which causes restrictions of ions hopping, which subsequently increases the resistance within the bulk of the material [66].

#### 3.3.4. Activation Energy (Ea) of Plasticized CPE

**Figure 12:**
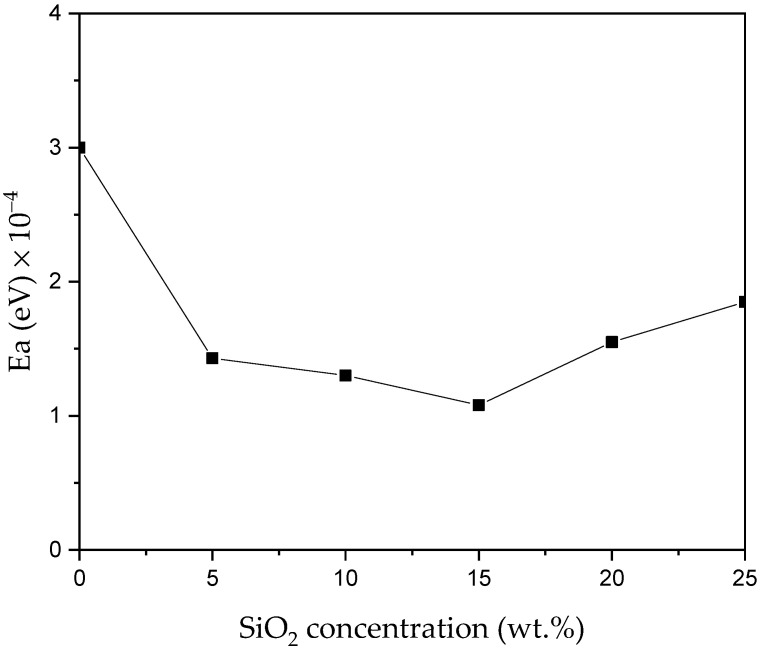
Activation energy (*E_a_*) versus SiO_2_ concentration.

To determine the ion dynamic of the prepared electrolytes further, activation energy (*E_a_*) was computed by fitting it in the Arrhenius equation (Equation (3)) [67]. Figure 12 illustrates the result of the activation energy as a function of SiO_2_ concentration. The results show that activation energy decreased significantly with the increase in SiO_2_ concentration up to 15 wt% (highest conducting electrolytes). However, with the incorporation of SiO_2_ content beyond 15 wt.%, the E_a_ suddenly increased. The sample with 15 wt.% SiO_2_ gave the highest conductivity, hence the sample with the lowest *E_a_* (5.51 × 10^−4^ eV) suggesting that PKS15 may have the highest ionic motion (hopping) [68]. As discussed earlier, addition of filler was found to disrupt the interaction between the polymer backbones [69]. Thus, it requires lesser energy to break and reform the coordination bond among the polymer matrix. As a result, the ion diffusion is more favorable and this enhances the ionic conductivity [70].

The result of Ea obtained in this study is in accordance with the previous studies. For instance, Wang and co-authors [71] reported that the highest conducting sample in all-solid-state electrolytes with a conductivity of 1.608 × 10^−4^ Scm^−1^ had the lowest activation energy (0.367 eV). This implies that the ions in highly conducting samples require lower energy for migration. Lower activation energy also resulted from the short distance between transit sites provided by the blended polymers. Table 4 presents the summary of activation energies for all the prepared CPEs.

#### 3.3.5. Electrochemical Stability

**Figure 13:**
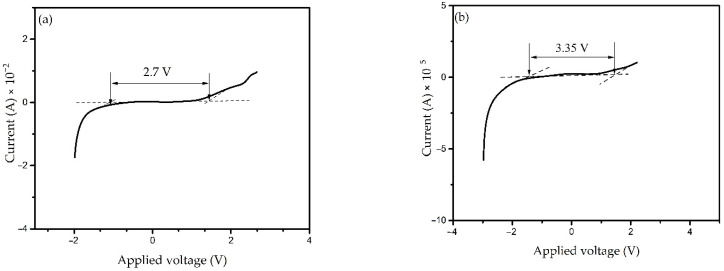
Linear sweep voltammetry (LSV) plots of the synthesized CPE (**a**) PKS0 and (**b**) PKS15 composites.

The electrochemical stability window (ESW) of the polymer electrolyte is a vital measure used in energy storage devices. The ESW of the PKS0 and of the highest conducting electrolyte (PKS15) at 5 mV s^−1^ scan rate is shown in Figure 13a,b. It was observed that the current remained constant with an increase in applied voltage until a definite limit (V_max_) was reached whereby the current suddenly increaseed rapidly for all electrolytes. This sudden current increment is linked to the decomposition of the polymer electrolyte [72,73]. It can be clearly observed that PKS15 electrolyte showed a wider electrochemical stability window of 3.35 V as compared to PKS0 at 2.7 V. This could be attributed to the inclusion of silica particles that enhanced the electrochemical stability of the electrolyte. According to reported study, electrochemical stability may be influenced owing to the influence of the silica and the dielectric constant of the host polymer, which would give a higher concentration of charge carriers [74]. SiO_2_ is known to be an effective ingredient that increases the amorphous phase of the electrolyte, which is attributed to the creation of mobile defects as well as an increase in the flow of the material possibly at the interface region. Moreover, the degree of amorphosity that predominantly controls the ion conduction in the polymer matrix could be greatly enhanced by the incorporation of fillers [75]. Therefore, incorporation of SiO_2_ to the polymer electrolyte significantly increased the potential window of the synthesized electrolyte in this study. Similarly, addition of SiO_2_ is known to increase the anodic stability of the electrolytes [76].

Based on this study, the PVA-K_2_CO_3_-SiO_2_ system is electrochemically stable up to 3.35 V, which is sufficient to allow safer application in next generation electrochemical devices. The result achieved in this study is higher than the minimum recommended standard electrochemical window (1.7 V) of an electrolyte material for EDLC applications of other studies earlier reported [77,78,79]. Therefore, the result demonstrates that the electrolyte synthesized and characterized in this study is suitable for fabrication in electrochemical devices.

## 4. Conclusions

In this study, various amounts of SiO_2_ particles as filler were incorporated into the non-filler PVA-K_2_CO_3_ composites to prepare composite polymer electrolytes (CPEs) with improved structural properties, ion transport mechanism, ionic conductivity, and potential window. The effect of SiO_2_ on the physical properties and ionic conductivity of PVA-K_2_CO_3_ composite was clearly noticeable. FESEM and DSC studies indicated that the increase in amorphous phase was relatively high after incorporation of filler (plasticization). This is due to the effect of SiO_2_ particles, which relaxed the backbone of the polymer and hence yielded bendable and stretchable polymer backbone that aided in stimulating ionic movement in the polymer matrix. The optimum electrochemical performance was exhibited by PKS15 (15 wt.% SiO_2_) with an optimum ionic conductivity of 7.86 × 10^−3^ mScm^−1^ at 373.15 K and a potential window of 3.35 V. The temperature-conductivity relationship was found to follow Arrhenius equation. The results obtained show that the use of SiO_2_ to plasticized PVA-K_2_CO_3_ electrolyte has a better prospective for application in portable and bendable next generation electrochemical devices.

## Figures and Tables

**Table 1:** Designation and composition of the synthesized composite polymer electrolytes (CPEs).

Designation	SiO_2_ Composition (wt.%)
PKS0	0
PKS5	15
PKS10	10
PKS15	15
PKS20	20
PKS25	25

**Table 2:** Summary peaks (cm^−1^) of the synthesized composite polymer electrolytes (PKS).

Description Vibration Modes	Wavenumber (cm^−1^)
Stretching of O–H of PVA	3258
Asymmetric C–H stretching of pure PVA	2941
C=O broadening	1647
Strong C=C broadening	1562
Winding vibration of CH_2_	1411
CH wagging vibrations	1141
C–O stretching of acetyl group	1090
C–H rocking	843
Free^-^ anions	657
Symmetric Si–O–Si stretch	917
Asymmetric Si–O–Si stretch	1332

**Table 3:** Electrochemical properties of the synthesized composite polymer electrolytes in comparison with previous studies at room temperature.

Electrolytes	Plasticizers	σ, (mScm^–^^1^)	Ref
PVA-K_2_CO_3_	SiO_2_	3.25 × 10^–4^	This study
Li_2_O−LiF−P_2_O_5_	MoO_3_	4.40 × 10^–7^	[61]
Lithium polystyrene sulfonate brushes (LiPSS)	SiO_2_	7.20 × 10^–4^	[61]
PVC	SiO_2_	5.0 × 10^–4^	[61]
PEO/PEG	KI	5.27 × 10^–5^	[58]
Starch-chitosan doped with NH_4_I	Glycerol	1.28 × 10^–3^	[53]
Starch–chitosan	NH_4_I	3.04 × 10^–4^	[59]
Chitosan-based solid biopolymer	NH_4_SCN/glycerol	1.51 × 10^–3^	[62]
Chitosan	NH_4_Br	2.15 × 10^–4^	[60]
Chitosan-PEO NH_4_NO_3_	Ethylene carbonate (EC)	2.06 × 10^–3^	[63]

**Table 4:** Activation energy (*E_a_*) of the synthesized CPEs based on PVA-K_2_CO_3_-SiO_2._

Samples	Activation Energy (*E_a_*) (eV)
PKS0	5.51 × 10^−4^
PKS5	1.43 × 10^−4^
PKS10	1.30 × 10^−4^
PKS15	1.09 × 10^−4^
PKS20	1.55 × 10^−4^
PKS25	1.85 × 10^−4^

## Data Availability

The data presented in this study are available on request from the corresponding author. The data are not publicly available due to privacy.
